# Supplementary material for: SiFT: uncovering hidden biological processes by probabilistic filtering of single-cell data
Source: Nat Commun. 2024 Jan 26;15:760. doi: 10.1038/s41467-024-44757-7 (PMC10817921; doi:10.1038/s41467-024-44757-7)
Supplement: Supplementary file 1 — Supplementary Information [file 41467_2024_44757_MOESM1_ESM.pdf]

SiFT: Uncovering hidden biological processes by probabilistic filtering of single-cell data

Supplementary Information

Zoe Piran[1] and Mor Nitzan[1,2,3*]

[1]School of Computer Science and Engineering, The Hebrew University, Jerusalem, Israel.
[2]Racah Institute of Physics, The Hebrew University, Jerusalem, Israel.
[3]Faculty of Medicine, The Hebrew University, Jerusalem, Israel.
[*]Correspondence to mor.nitzan@mail.huji.ac.il

Table of Contents

Supplementary Figures **2**

Supplementary Figure 1 2
Supplementary Figure 2 3
Supplementary Figure 3 4
Supplementary Figure 5 6
Supplementary Figure 6 7
Supplementary Figure 7 8
Supplementary Figure 8 9
Supplementary Figure 9 10
Supplementary Figure 10 11
Supplementary Figure 11 11
Supplementary Figure 12 12
Supplementary Figure 13 13

Supplementary Tables **14**

Supplementary Table 1 16
Supplementary Table 2 17

Supplementary References **18**

Supplementary Figures

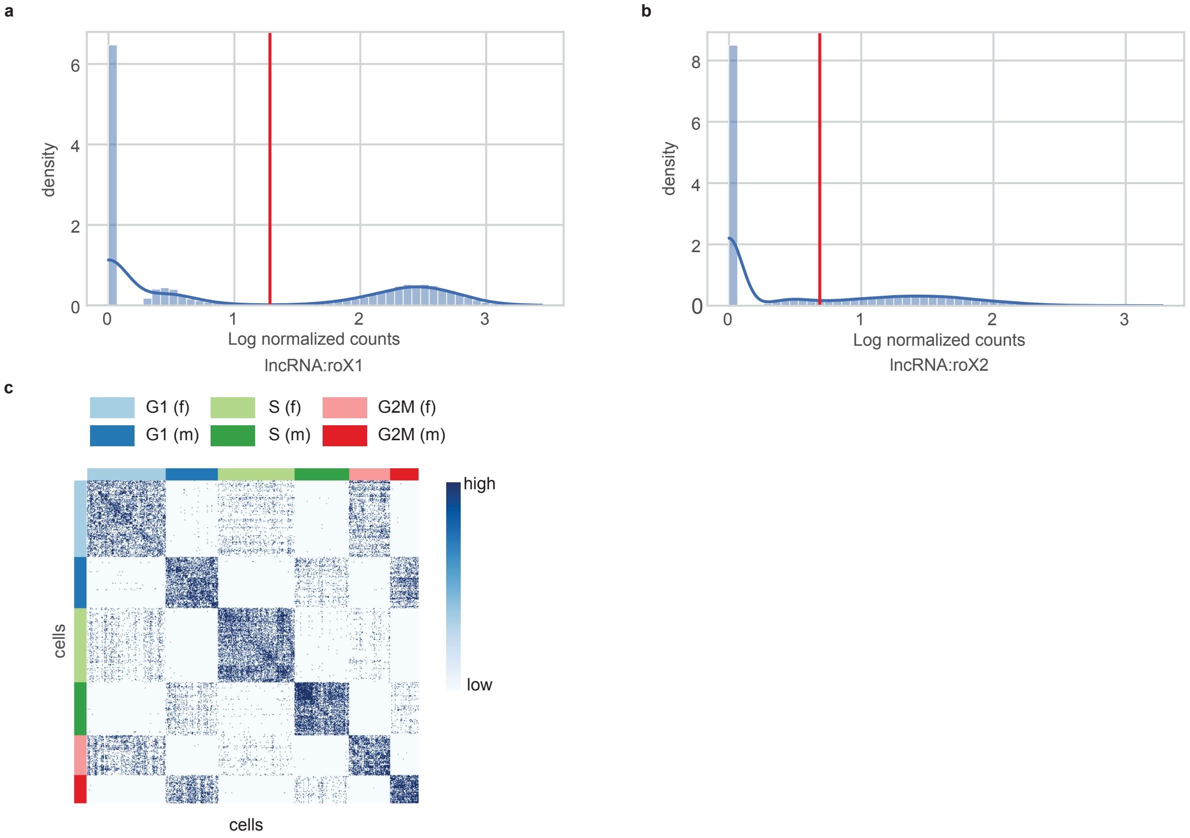

Supplementary Figure 1: Pre-processing for the removal of unwanted sex and cell cycle effects from single-cell transcriptomics of the Drosophila wing disc development[1]. **a, b** Probability histogram plots of the log normalized expression counts for **a** *lncRNA:roX1* and **b** *lncRNA:roX2* within all cells. Density curves for the data are shown in blue. Red lines are drawn on the first local minima within the density of the data and serve as a cutoff for classifying cells as having a high or low expression of either gene (cutoffs at 1.28 (a) and 0.69 (b)). Cells with high expression of either *lncRNA:roX1 or lncRNA:roX2* were classified as male-originating; otherwise, cells were designated as female-originating. **c** The SiFT kernel, a knn kernel based on the graph connectivity matrix based on the set of sex and cell cycle genes. Cells are ordered according to the combined label of sex and cell cycle phase.

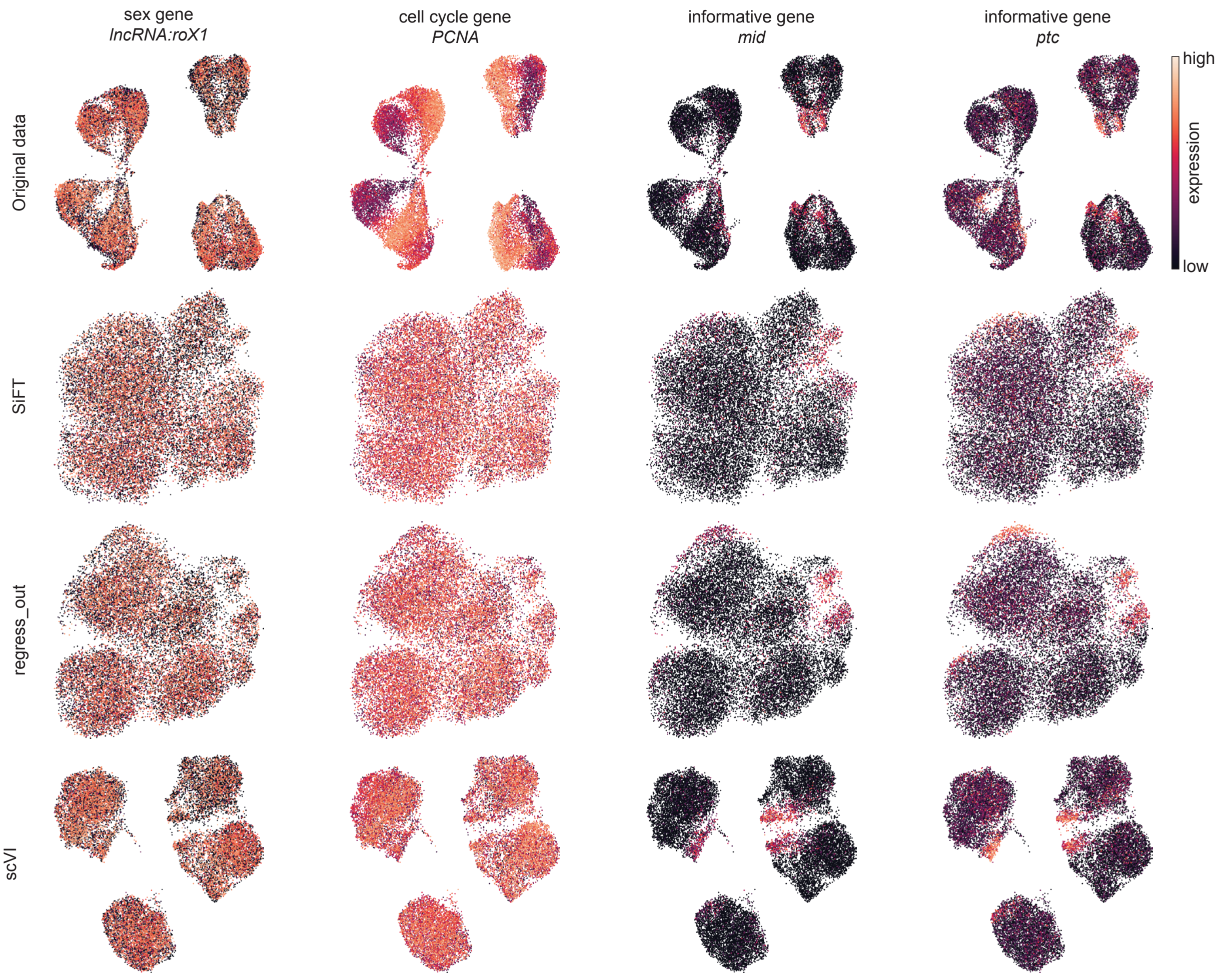

Supplementary Figure 2: removal of unwanted sex and cell cycle effects from single-cell transcriptomics of the Drosophila wing disc development. UMAP embeddings following different data correction procedures (rows) and colored by genes representing different data features (columns). Rows (top to bottom) show uncorrected data (Original data), SiFT filtered (SiFT), Scanpy's (scanpy.pp.regress_out(), regress_out), and scVI latent space with continuous covariates correction (scVI). Columns (left to right) *lncRNA:roX1* (sex gene), *PCNA* (cell cycle gene), *mid* and *ptc* (informative genes reported by[1]).

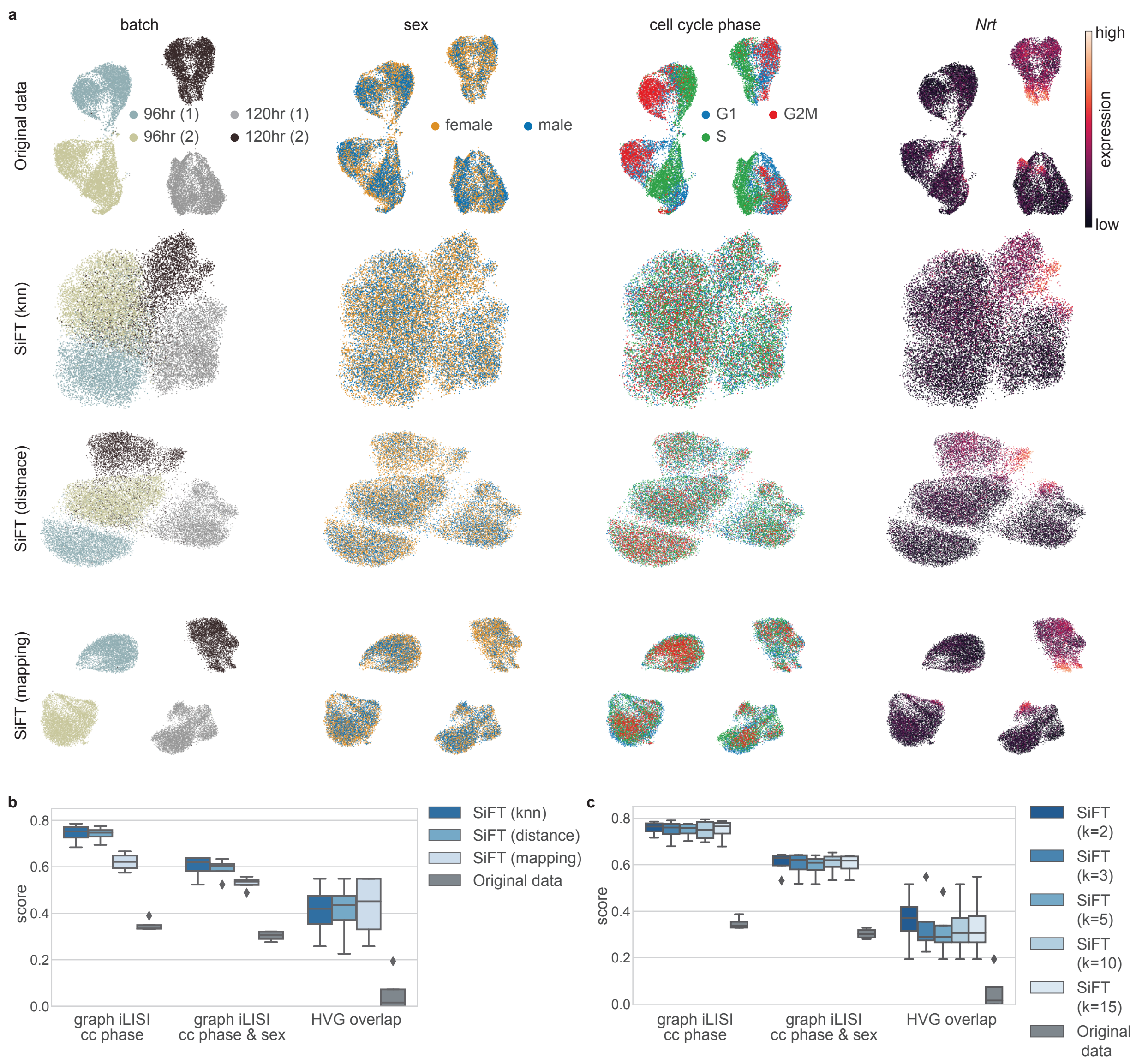

Supplementary Figure 3: different SiFT kernels applied to single-cell transcriptomics of the Drosophila wing disc development[1]. **a** UMAP embeddings following different SiFT kernels procedures (rows) and colored by different covariates of unwanted sources of variation (columns). Rows (top to bottom) show uncorrected data (Original data), data filtered by SiFT using knn kernel (SiFT (knn)), distance kernel (SiFT (distance)), and a mapping kernel (SiFT (mapping)). Columns (left to right) show the batch label, sex label, cell cycle phase, and cell cycle and sex. **b** The graph iLISI and hvg score for each data correction procedure obtained for the different covariate labels. **c** The graph iLISI and hvg score for different choices of the number of neighbors (k) considered for the knn kernel (Methods). In (b)-(c) scores are reported over $n = 4$ biologically independent samples. Middle line in box plots, median; box boundary, interquartile range (IQR); whiskers, 1.5*IQR; minimum and maximum, not indicated in the box plot; gray dots, points beyond the minimum or maximum whisker. Source data of (**b, c**) are provided as a Source Data file.

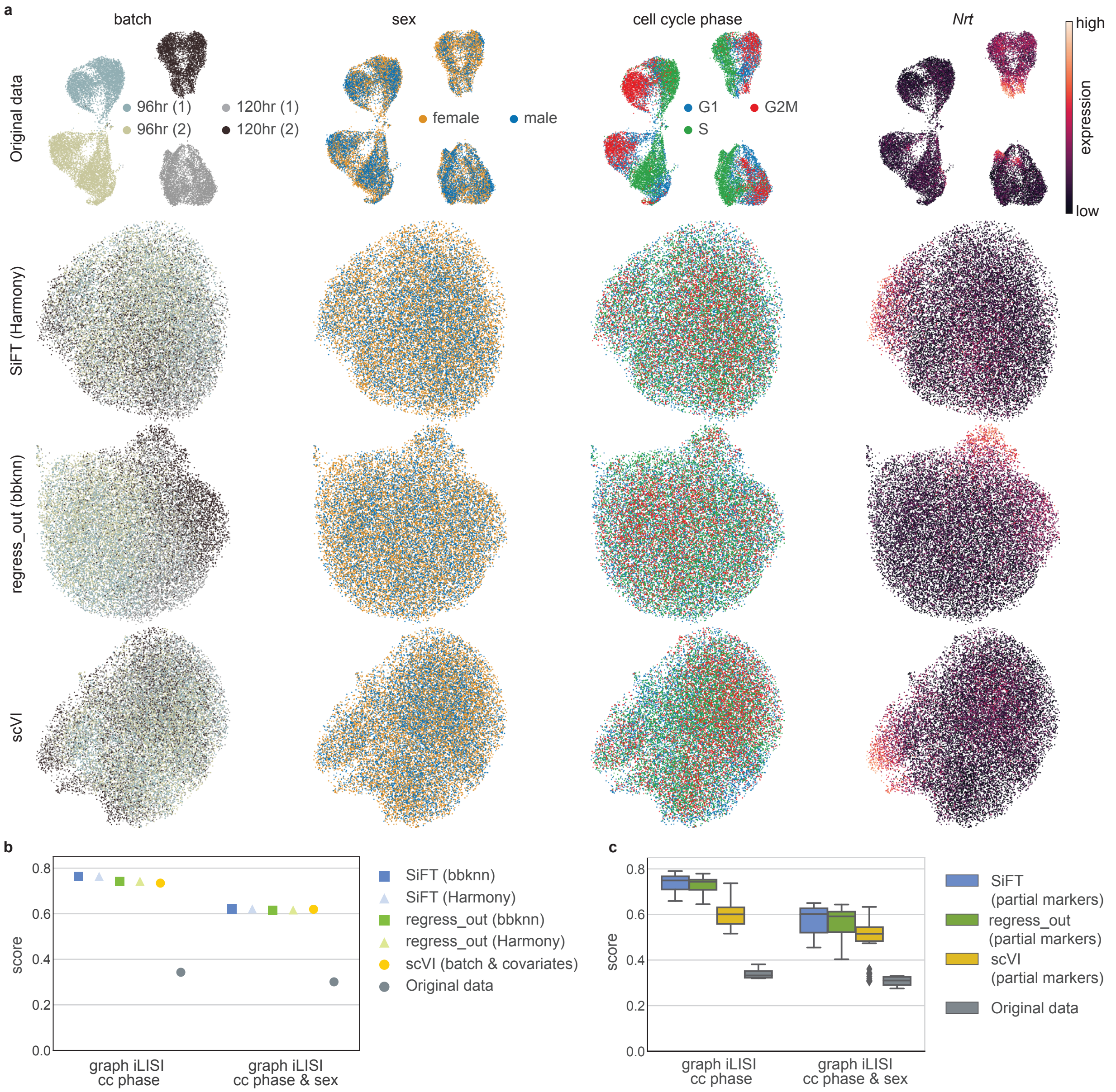

Supplementary Figure 4: Benchmarking performance and robustness for the removal of unwanted variation from the Drosophila wing disc development data[1]. **a, b** Combining batch integrations with the removal of unwanted sources of variation. **a** UMAP embeddings following different data correction procedures which include batch integration as well as the removal of unwanted sources of variation (rows) and colored by different covariates of unwanted sources of variation (columns). Rows (top to bottom) show uncorrected data (Original data), SiFT filtered followed by Harmony integration (SiFT (harmony)), regress_out followed by bbknn (regress_out (bbknn)), and scVI latent space with batch and continuous covariates (scVI (batch & cov.)). Columns (left to right) show the batch label, sex label, cell cycle phase, and cell cycle and sex (Methods). **b** The graph iLISI score each data correction procedure obtained for the different covariate labels. **c** Application of the different methods using partial markers. We modify the set of marker genes (10 times) by randomly sampling 10 of the 55 marker genes (sex and cell cycle) and

replacing them with 10 random genes from the set which is defined as - genes of interest, implying the original marker genes are replaced with genes encoding desired biological information we wish to retain post-filtering. That is, we weaken the set of markers by not only removing genes that encode the unwanted variation but also adding genes that necessarily contain biological signals that are of interest for post-filtering downstream analysis (Methods, Supplementary Table 1). We then evaluate all methods using the same modified, 10 random gene sets, and report the graph iLISI score obtained for the different covariate labels. In comparison to the scores obtained using the original marker set we observe the following percentage of deviation of the mean score (taken over biological batches and modified marker set repetition): iLISI cell cycle; SiFT=-0.6%, regress_out=-1.0%, scVI=-13.5%, iLISI cell cycle and sex; SiFT=-4.2%, regress_out=-5.9%, scVI=-16.3%. scores are reported over $n = 40$ points based on the the 10 different partial marker sets biologically and 4 biological independent samples. Middle line in box plots, median; box boundary, interquartile range (IQR); whiskers, 1.5*IQR; minimum and maximum, not indicated in the box plot; gray dots, points beyond the minimum or maximum whisker. Source data of (**b, c**) are provided as a Source Data file.

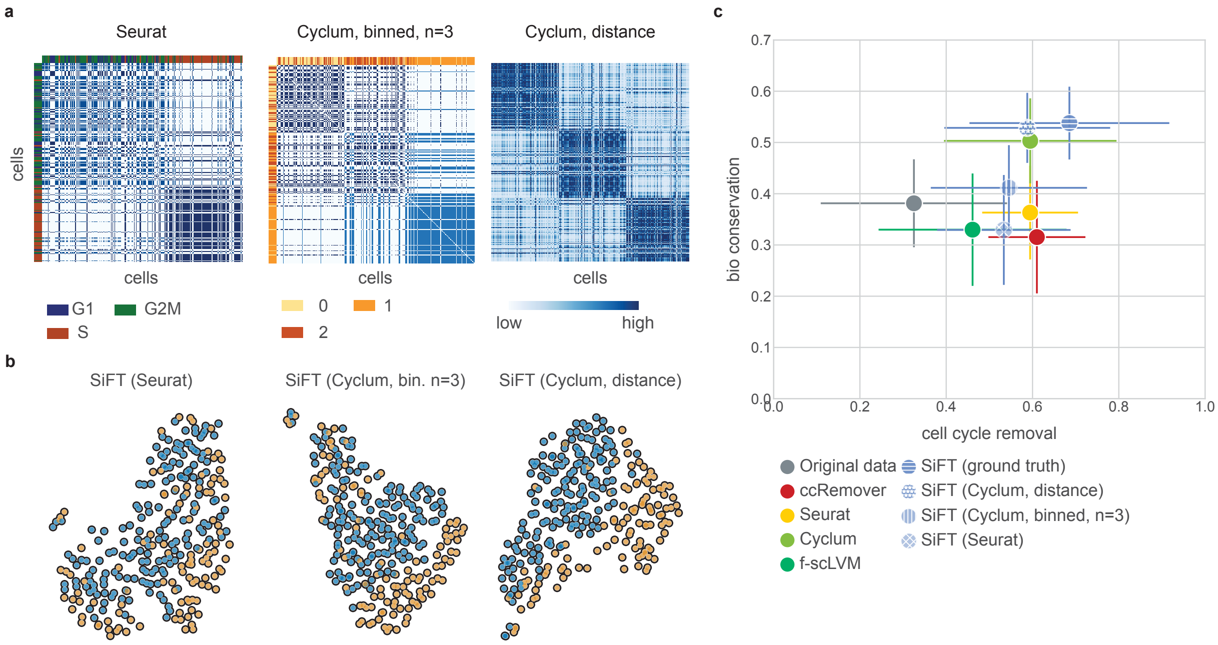

Supplementary Figure 5: Filtering the cell-cycle effects from the virtual tumor data consisting of two subclones[2]. **a** Different cell-cell similarity kernels are defined by SiFT. Cells are ordered according to the ground truth cell cycle stage. (left) a mapping kernel based on Seurat cell cycle stage classification. The row(col) colors depict the Seurat inferred cell cycle stage. (center) a mapping kernel based on binning of the Cyclum pseudotime (n, number of bins, $n = 3$), the row(col) colors depicts the cells' bin. (right) a distance kernel distances defined over the Cyclum pseudotime prediction. **b** UMAP of the filtered data colored by the sub-clone identity. (left) Seurat cell cycle stage prediction (center) Cyclum binning ($n = 3$) (right) distance in Cyclum pseudotime. **c** Scatter plot of the mean overall bio conservation score against mean overall cell cycle removal scores using the metrics defined in[3] (Methods). Error bars indicate the

mean standard error considering $n = 6$ bio conservation metrics (y-axis) and $n = 4$ cell cycle removal metrics (x-axis). Source data of (c) are provided as a Source Data file.

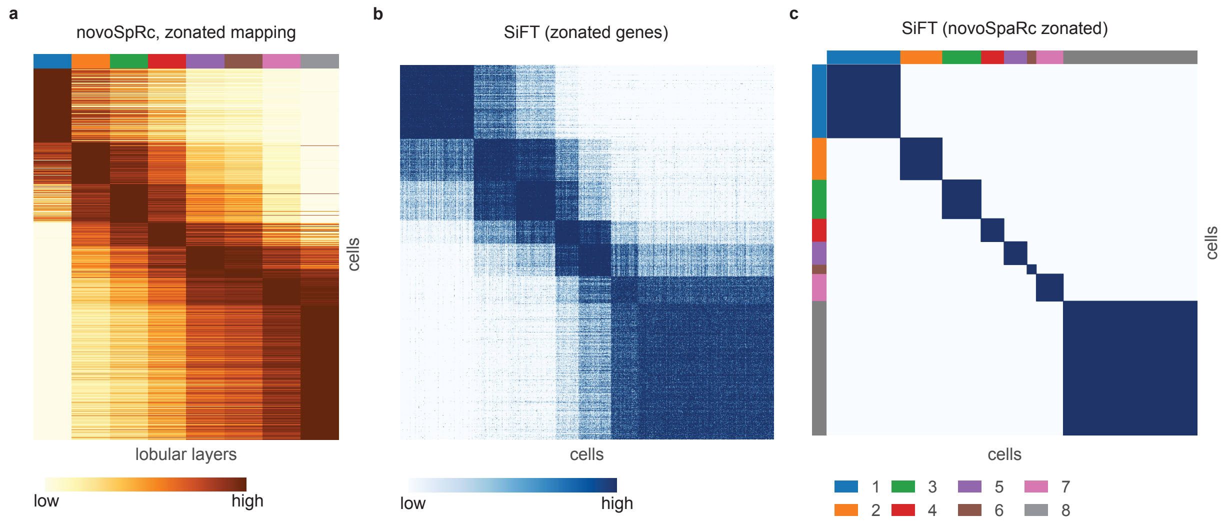

Supplementary Figure 6: Enhancing circadian clock signal in the mammalian liver[4]. **a** The mapping of the mammalian liver to eight lobular layers as obtained by novoSpaRc[5,6]. **b, c** The SiFT kernels used for filtering; **b** zonated genes-based kernel and **c** novoSpaRc zonation reconstruction-based kernel**.**

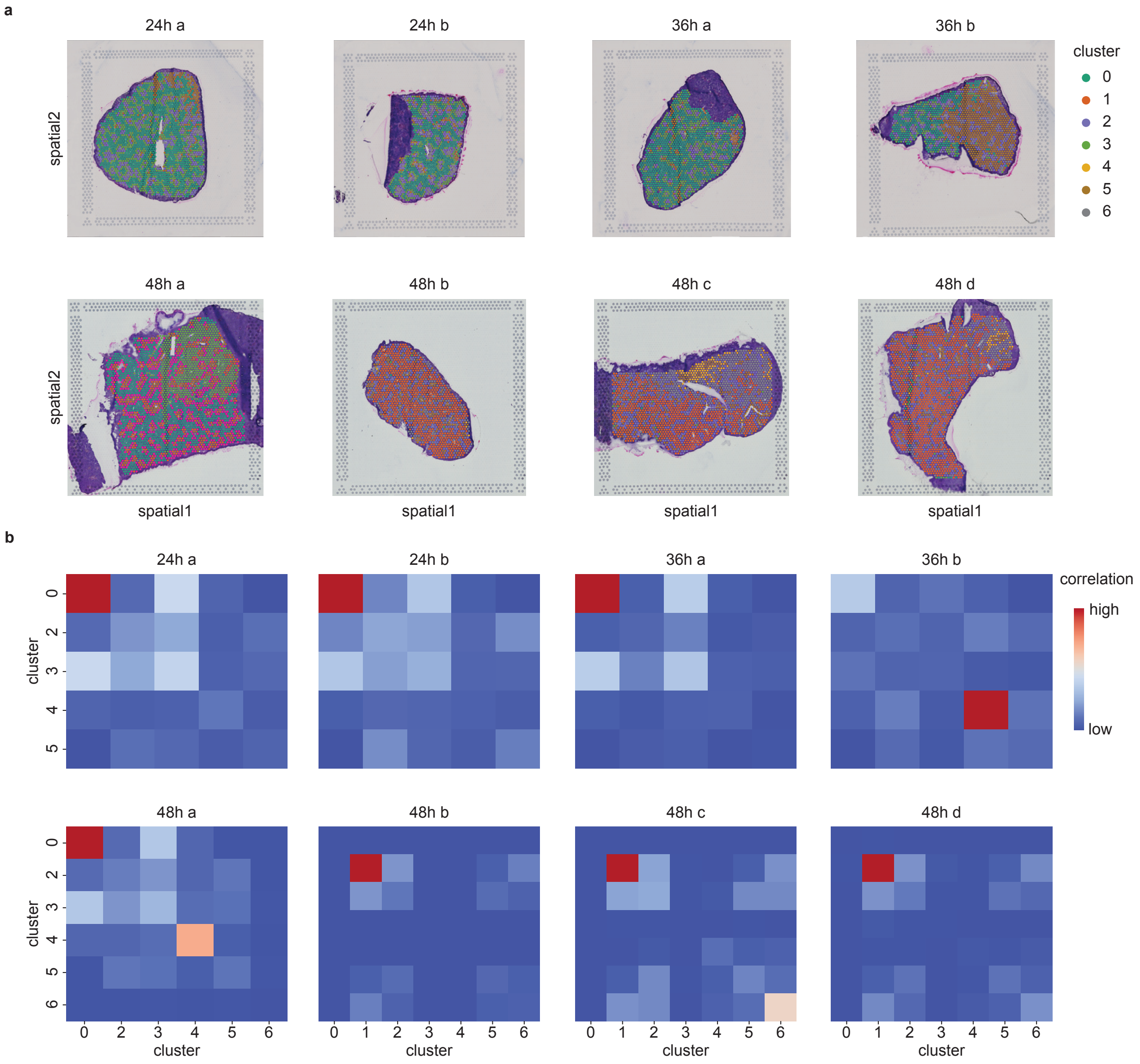

Supplementary Figure 7: Analysis of spatial transcriptomics (ST) samples of liver regeneration post APAP-induced liver injury in mice[7]. **a** Spatial visualization of leiden clustering results of the original data after integration of cells using Harmony. Subplots represent different samples taken at the different time points, 24, 36, and 48 hours post injury. **b** The clusters' interaction matrix evaluated over the spatial correlation of the leiden clusters within each sample (Methods)

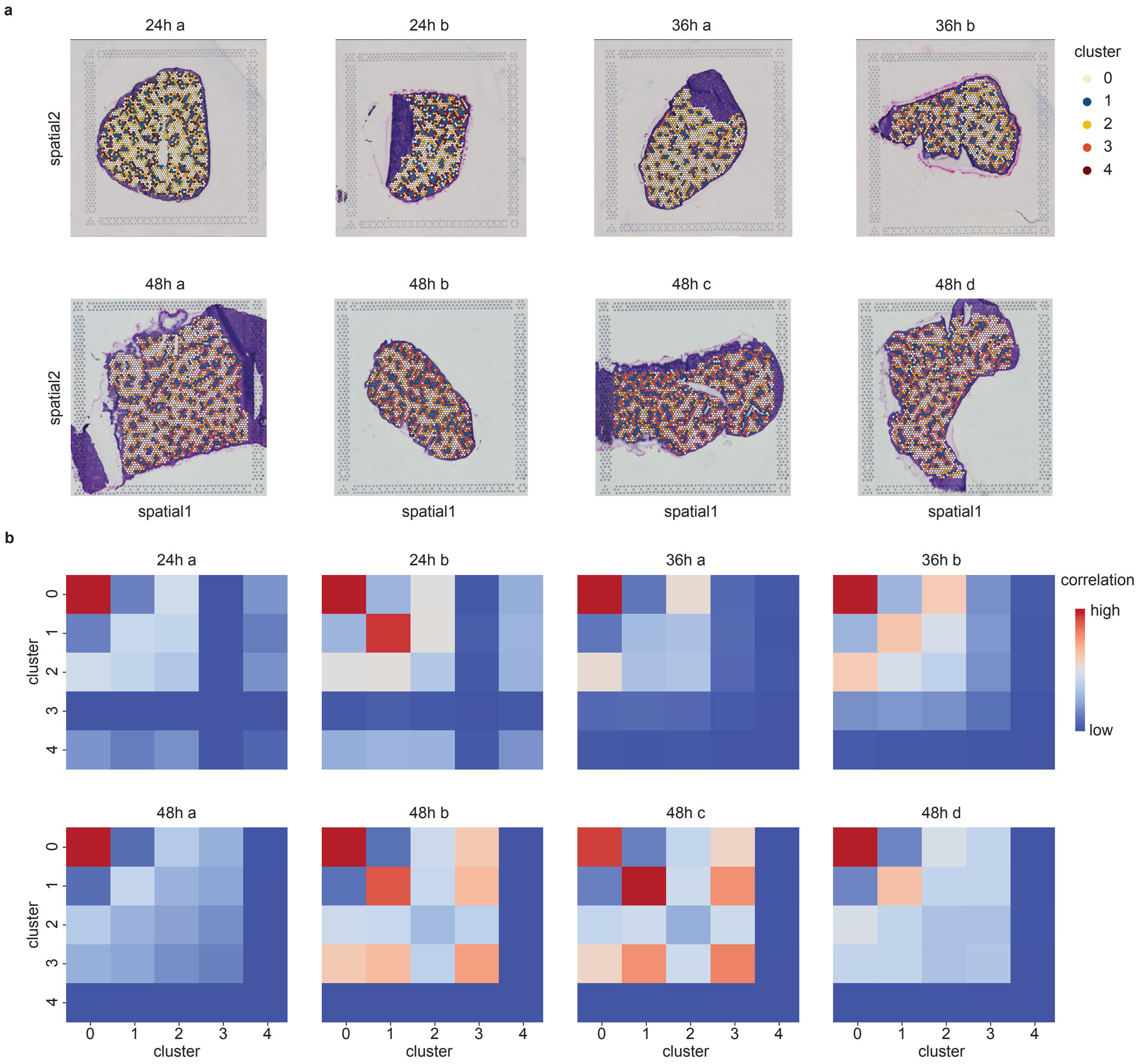

Supplementary Figure 8: Analysis of SiFT filtered representation of ST samples of liver regeneration post APAP-induced liver injury in mice[7]. **a** Spatial visualization of leiden clustering results of the SiFTed data. Subplots represent different samples taken at the different time points, 24, 36, and 48 hours post injury. **b** The clusters' interaction matrix evaluated over the spatial correlation within each sample (Methods).

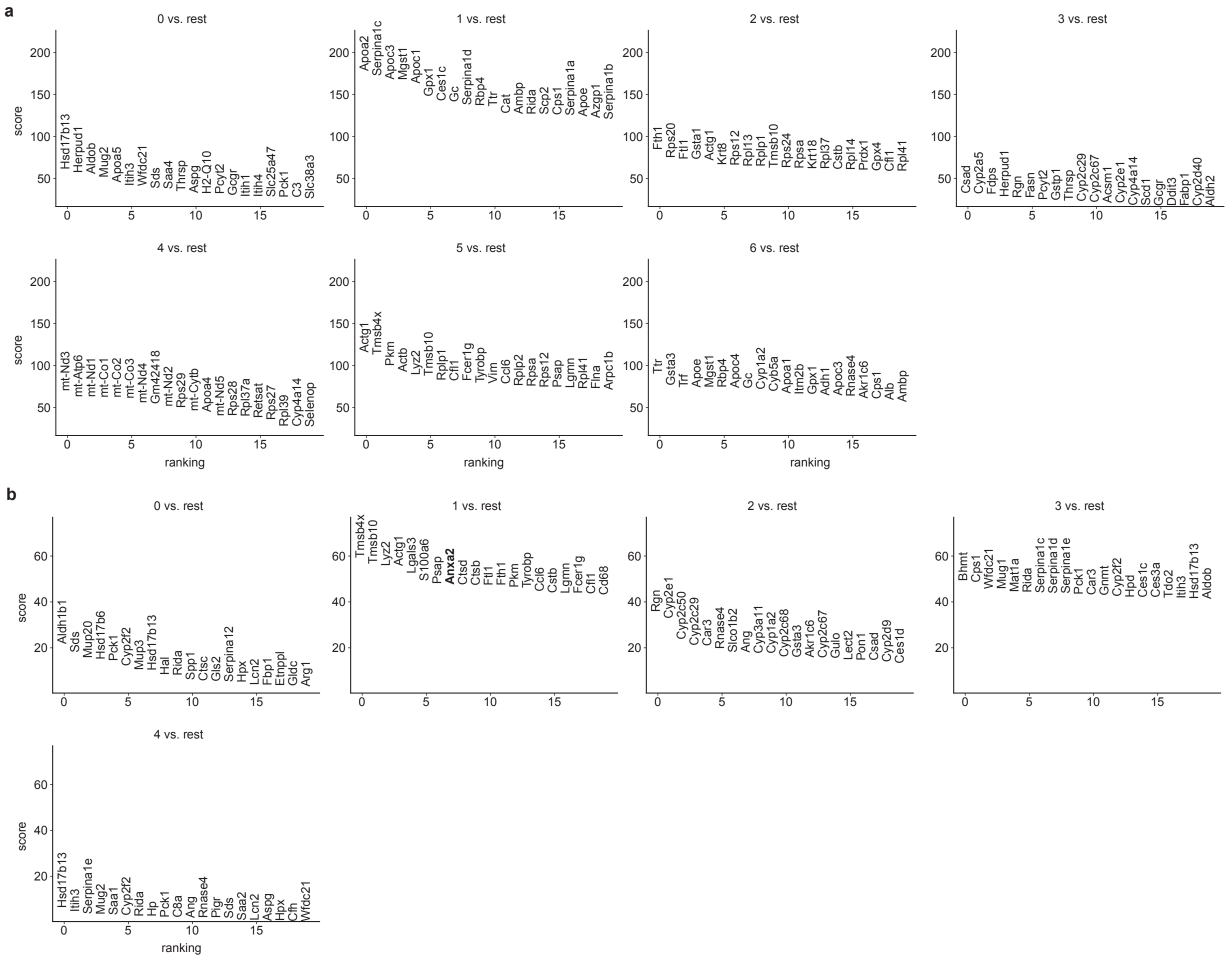

Supplementary Figure 9: a, b Differentially expressed genes between the different leiden clusters found in the analysis ST samples of liver regeneration post APAP-induced liver injury in mice[7]. Results are over leiden clusters in **a** the original data and **b** the SiFTed data.

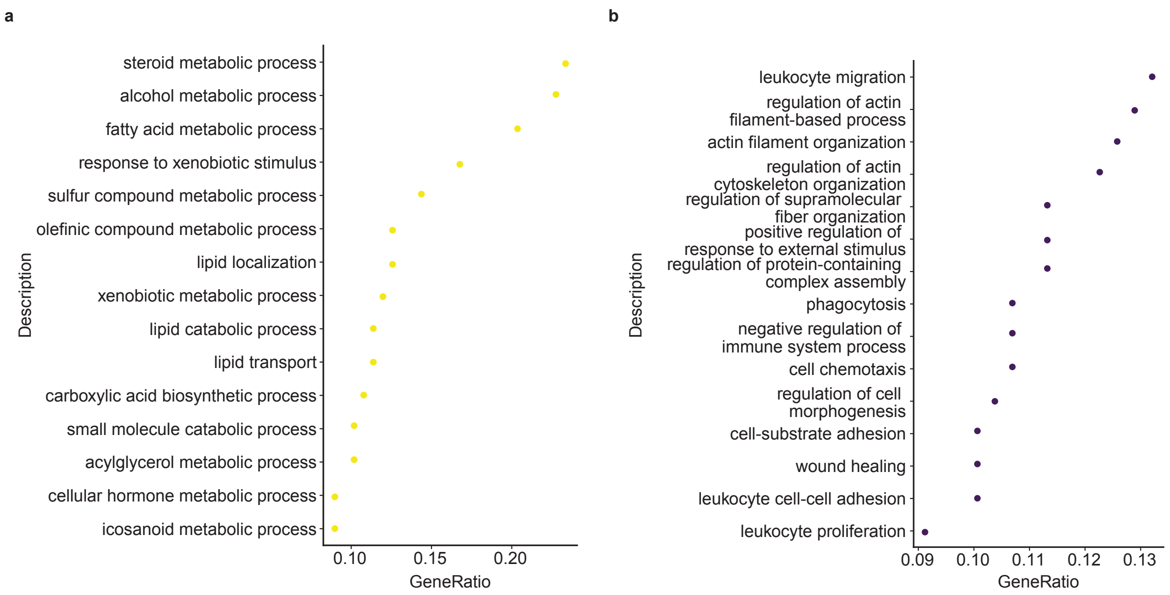

Supplementary Figure 10: Pseudotime analysis of the migratory hepatocyte subpopulation found using SiFT in ST samples of liver regeneration post APAP-induced liver injury in mice[7]. **a, b** Enrichment results over genes overexpressed in cells assigned to **a** early and **b** late pseudotime.

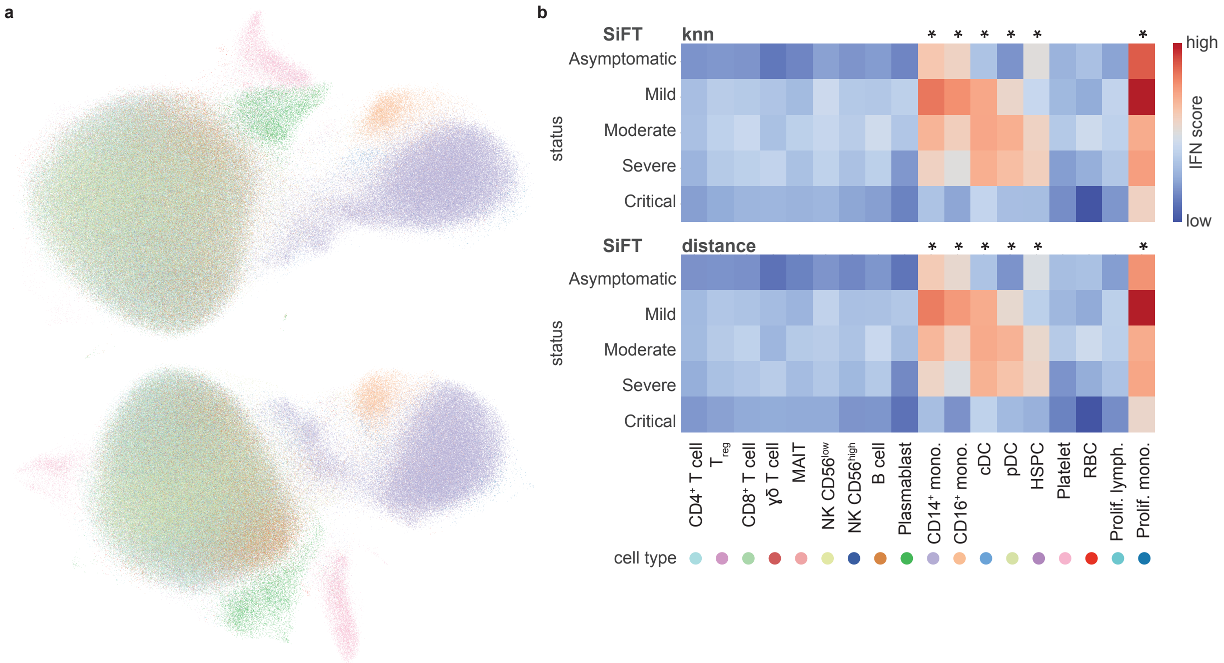

Supplementary Figure 11: Robustness to kernel choice in the application of SiFT to the COVID-19 dataset[8]. **a** UMAP visualizations of cells in the data after applying SiFT with knn kernel (top) and distance kernel (bottom) colored by cell type. **b** Enrichment of interferon response of each cell state separated by disease severity. Shown for SiFT with knn kernel (top) and *distance* kernel (bottom). IFN response was calculated using a published gene list (GO:0034340). Statistical tests were performed with a one-sided Mann-

Whitney U test between the cell types. Cell types are considered statistically significant if $p_{val} < 0.05$ (denoted by *). Source data of (b) are provided as a Source Data file.

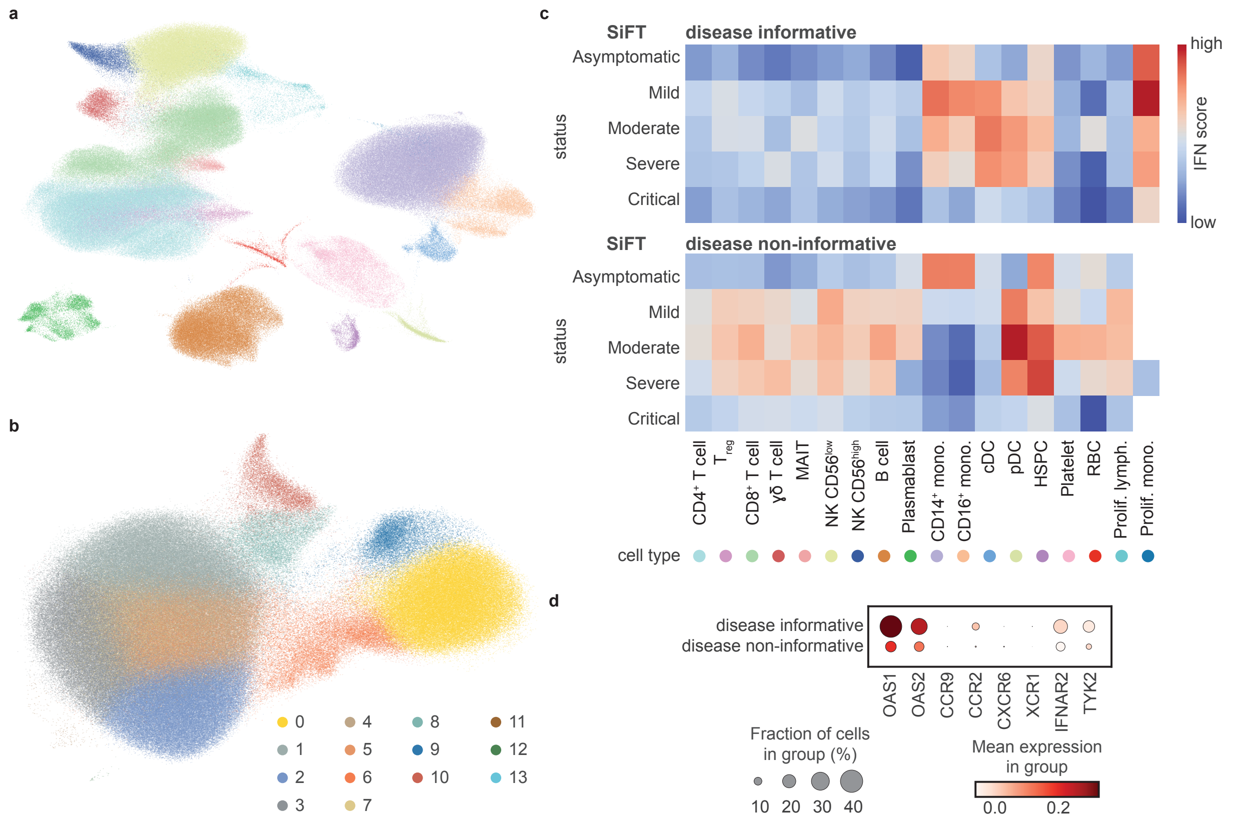

Supplementary Figure 12: Revealing the disease signal in COVID-19 dataset[8]. **a** UMAP visualizations of cells in the original data colored by reported cell types. **b** UMAP visualizations of cells in the data after applying SiFT, colored by Leiden clustering of the SiFTed data. **c** Enrichment of interferon response of each cell state separated by disease severity. Shown for SiFTed disease informative cells (top) and SiFTed disease non-informative cells (bottom). IFN response was calculated using a published gene list (GO:0034340). **d** Dot plot of gene expression where the color is scaled by mean expression and the dot size is proportional to the percent of the population expressing the gene considering genes associated with COVID-19 identified in recent GWAS studies[9,10].

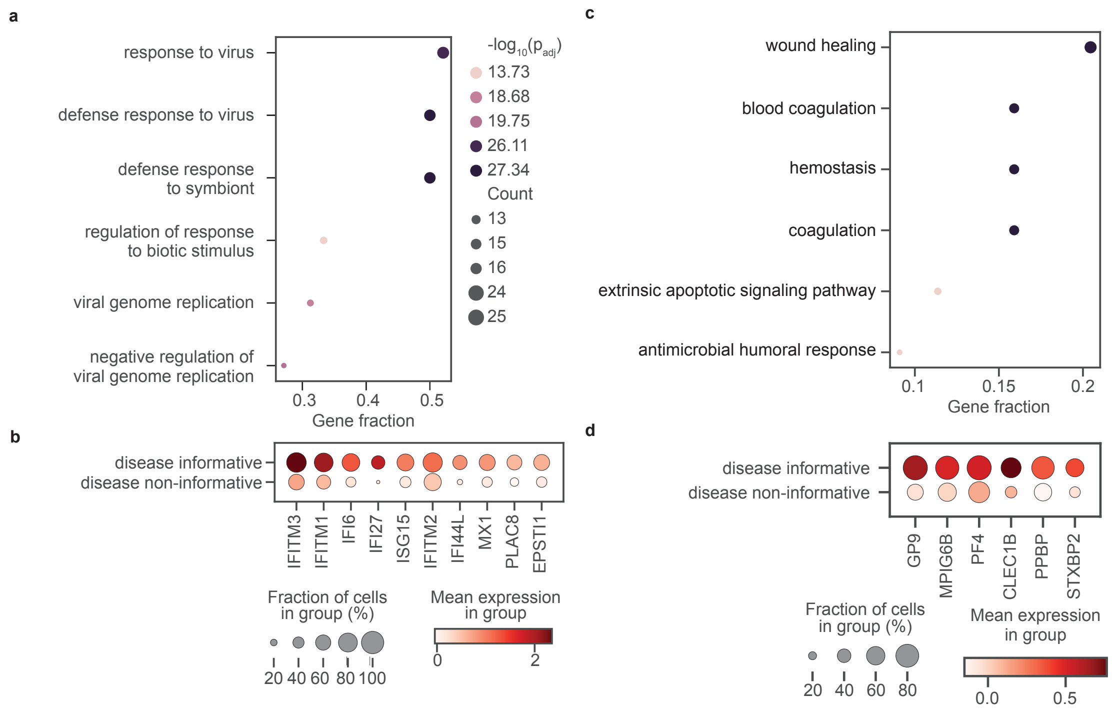

Supplementary Figure 13: Analysis of specific cell types and their disease associated signal in COVID-19 dataset[8]. **a, b** Analysis of cDC disease informative cells (compared to non-informative). **a** Enrichment of differentially expressed genes. **b** Dot plot of gene expression of differentially expressed genes. **c, d** Analysis of Platelet disease informative cells (compared to non-informative). **c** Enrichment of differentially expressed genes. **d** Dot plot of gene expression of platelets activation markers in the. Markers taken from[8]. In (**a, c**) size of circles indicates the number of genes color indicates the magnitude of $-log_{10}(p_{adj})$. p_{adj} is calculated using a permutation test with Benjamini-Hochberg correction. Source data of (**a, c**) are provided as a Source Data file.

Supplementary Tables

Sex genes	Cell cycle genes	Genes of interest
lncRNA:roX1	PCNA	Argk
lncRNA:roX2	dnk	Nrt
Sxl	RnrS	Ten-a
msl-2	RnrL	Ten-m
	Claspin	wb
	Mcm5	Act57B
	Caf1-180	drl
	RPA2	mid
	HipHop	nemy
	stg	lms
	Mcm6	CG11835
	dup	Gyg
	WRNexo	ara
	Mcm7	tok
	dpa	kirre
	CG10336	NK7.1
	Mcm3	fj
	Mcm2	beat-IIIc
	RpA-70	CG33993
	Chrac-14	dpr16
	CG13690	CG15529
	RPA3	CG9593
	asf1	beat-IIb
	CDC45L	robo2

	DNApol-alpha73	Ama
	CycE	fz2
	DNApol-alpha50	elB
	Kmn1	noc
	Lam	nkd
	Nph	fng
	msd5	vg
	msd1	
	ctp	
	Set	
	scra	
	Chrac-16	
	ncd	
	Ote	
	pzg	
	HDAC1	
	nesd	
	tum	
	CG8173	
	aurB	
	feo	
	pav	
	CG6767	
	sip2	
	Det	
	Cks30A	

	CycB	
	B52	

Supplementary Table 1. Gene sets for multiple covariates Drosophila analysis (based on[11]).

Spatial Genes	Temporal genes
Glul	Bmal1 (Arntl)
Ass1	Clock
Asl	Npas2
Cyp2f2	Nr1d1
Cyp1a2	Nr1d2
Pck1	Per1
Cyp2e1	Per2
Cdh2	Cry1
Cdh1	Cry2
Cyp7a1	Dbp
Acly	Tef
Alb	Hlf
Oat	Elov3
Aldob	Rora
Cps1	Rorc

Supplementary Table 2. Spatial and temporal gene sets used in the analysis of the liver dataset (based on[4]).

Supplementary References

1. Everetts, N. J., Worley, M. I., Yasutomi, R., Yosef, N., & Hariharan, I. K. (2021). Single-cell transcriptomics of the Drosophila wing disc reveals instructive epithelium-to-myoblast interactions. *Elife*, *10*, e61276.
2. Liang, S., Wang, F., Han, J., & Chen, K. (2020). Latent periodic process inference from single-cell RNA-seq data. *Nature communications*, *11*(1), 1441.
3. Luecken, M. D., Büttner, M., Chaichoompu, K., Danese, A., Interlandi, M., Müller, M. F., ... & Theis, F. J. (2022). Benchmarking atlas-level data integration in single-cell genomics. *Nature methods*, *19*(1), 41-50.
4. Droin, C., Kholtei, J. E., Bahar Halpern, K., Hurni, C., Rozenberg, M., Muvkadi, S., ... & Naef, F. (2021). Space-time logic of liver gene expression at sub-lobular scale. *Nature metabolism*, *3*(1), 43-58.
5. Moriel, N., Senel, E., Friedman, N., Rajewsky, N., Karaiskos, N., & Nitzan, M. (2021). NovoSpaRc: flexible spatial reconstruction of single-cell gene expression with optimal transport. *Nature protocols*, *16*(9), 4177-4200.
6. Nitzan, M., Karaiskos, N., Friedman, N., & Rajewsky, N. (2019). Gene expression cartography. *Nature*, *576*(7785), 132-137.
7. Matchett, K. P., Wilson-Kanamori, J. W., Portman, J. R., Kapourani, A., Fercoq, F., May, S., ... & Henderson, N. C. (2023). Multimodal decoding of human liver regeneration. *bioRxiv*, 2023-02.
8. Stephenson, E., Reynolds, G., Botting, R. A., Calero-Nieto, F. J., Morgan, M. D., Tuong, Z. K., ... & Haniffa, M. (2021). Single-cell multi-omics analysis of the immune response in COVID-19. *Nature medicine*, *27*(5), 904-916.
9. Severe Covid-19 GWAS Group, Ellinghaus, D., Degenhardt, F., Bujanda, L., Buti, M., Albillos, A., Invernizzi, P., Fernández, J., Prati, D., Baselli, G., Asselta, R., Grimsrud, M. M., Milani, C., Aziz, F., Kässens, J., May, S., Wendorff, M., Wienbrandt, L., Uellendahl-Werth, F., Zheng, T., … Karlsen, T. H. (2020). Genomewide Association Study of Severe Covid-19 with Respiratory Failure. *The New England journal of medicine*, *383*(16), 1522–1534.
10. Pairo-Castineira, E., Clohisey, S., Klaric, L., Bretherick, A. D., Rawlik, K., Pasko, D., ... & Princess of Wales Hospital, Llantrisant, UK Sathe S. 133 Davies E. 133 Roche L. 133. (2021). Genetic mechanisms of critical illness in COVID-19. *Nature*, *591*(7848), 92-98.
11. Gayoso, A., Lopez, R., Xing, G., Boyeau, P., Valiollah Pour Amiri, V., Hong, J., … & Yosef, N. (2022). A Python library for probabilistic analysis of single-cell omics data. *Nature biotechnology*, *40*(2), 163-166.
